# Antiphospholipid Antibody Persistent Positivity Is Associated with Worsened Endothelial Dysfunction in Hemodialysis Patients: A Cross-Sectional Study

**DOI:** 10.3390/jcm14176115

**Published:** 2025-08-29

**Authors:** Maxime Taghavi, Saleh Kaysi, Lila Bekkai, Ghita Debbarh, Lucas Jacobs, Edouard Cubilier, Anne Demulder, Frédéric Collart, Marie-Hélène Antoine, Joëlle Nortier

**Affiliations:** 1 Nephrology and Dialysis Department, Brugmann University Hospital, Université Libre de Bruxelles (ULB), 1020 Brussels, Belgium; saleh.kaysi@chu-brugmann.be (S.K.); lila.bekkai@ulb.be (L.B.); ghita.debbarh@ulb.be (G.D.); lucas.jacobs@chu-brugmann.be (L.J.); edouard.cubilier@chu-brugmann.be (E.C.); frederic.collart@chu-brugmann.be (F.C.); joelle.nortier@chu-brugmann.be (J.N.); 2 Laboratory of Experimental Nephrology, Department of Biochemistry, Faculty of Medicine, Université Libre de Bruxelles (ULB), 1070 Brussels, Belgium; marie-helene.antoine@ulb.be; 3 Laboratory of Hematology and Haemostasis LHUB-ULB, Brugmann University Hospital, Université Libre de Bruxelles (ULB), 1020 Brussels, Belgium; anne.demulder@chu-brugmann.be

**Keywords:** antiphospholipid antibodies, endothelial dysfunction, flow-mediated dilation, hemodialysis, nitric oxide

## Abstract

**Introduction**: Endothelial dysfunction is a common feature of end-stage kidney disease, requiring hemodialysis (HD) and antiphospholipid antibody (aPL) persistent positivity. Endothelial dysfunction can be assessed with noninvasive tests such as flow-mediated dilation (FMD). In the HD population, it is not known whether aPL persistent positivity is associated with a more severe endothelial dysfunction. **Methods**: We performed a cross-sectional study in our HD patients. The FMD of the brachial artery was measured in 17 aPL-positive patients who fulfilled the inclusion criteria and were matched to 17 controls according to age, gender, diabetes mellitus, smoking status and markers of dialysis adequacy (Kt/V). **Results**: FMD was significantly lower in the aPL group with a mean of 6.9% and 11.8% in the aPL-positive and the control groups, respectively (mean difference (IC 95%): −4.9 (−8.3; −1.6), *p* = 0.006). aPL was associated with a higher c-reactive protein level, and longer HD vintage. There was no statistical difference between groups in terms of pre-dialysis urea and urinary output, dialysis adequacy (Kt/V), and history of cardiovascular disease or treatments. **Conclusions**: aPL persistent positivity in HD patients was associated with worse endothelial dysfunction, reflected by FMD measurements. These findings have to be confirmed in larger studies.

## 1. Introduction

Endothelium has a major role in maintaining the homeostasis of vascular tone by finely adapting the balance between vasoconstriction and vasodilation. Endothelial dysfunction is characterized by a disturbance in the balance between relaxing (i.e., prostacyclin, nitric oxide (NO)) and contracting factors (Prostaglandin H2 (PGH2), endothelin-1), favoring vasoconstriction [1]. Endothelial dysfunction has been associated with chronic kidney disease and end-stage kidney disease (ESKD), hypertension, heart failure, aging, diabetes mellitus, atherosclerosis, and vascular disease, as well as autoimmune diseases such as antiphospholipid syndrome [2,3,4,5,6]. Flow-mediated dilation (FMD) is a noninvasive method of assessing endothelial-dependent vasodilatation. It uses ultrasound imaging for the evaluation of post-occlusive reactive hyperaemia, usually of the brachial artery. This phenomenon is solely mediated by the NO in the brachial artery, therefore FMD is considered as a gold standard noninvasive evaluation of NO-mediated endothelial dysfunction [7,8].

Antiphospholipid syndrome (APS) is an autoimmune disorder characterized by the persistent positivity of at least one antiphospholipid antibody (aPL) along with thrombotic or non-thrombotic manifestations described in the 2023 ACR/EULAR classification criteria [9]. Endothelial dysfunction plays a central role in APS pathogenesis [6,10,11]. It is mainly explained by the inhibition of endothelial NO synthase (eNOS) shown to be dependent upon protein phosphatase 2A (PP2A), β2-glycoprotein I (β2GPI), and apolipoprotein E receptor 2 [6], whereas other mechanisms have been described [6,12,13,14]. This will result in an increased risk of thrombosis, accelerated atherosclerosis and cardiovascular events in APS patients [12,15].

APS and aPL persistent positivity without APS are frequent in hemodialysis (HD) patients, and have been associated with HD vascular access complications such as arteriovenous fistula (AVF) thrombosis, stenosis and maturation failure, as well as intrastent restenosis [16,17,18,19,20]. However, the pathophysiology of those complications is not well understood and endothelial dysfunction may play a crucial role. In HD patients, the possible additional involvement of aPL on top of uremia in the pathophysiology of endothelial dysfunction has never been investigated.

The aim of the present study is to investigate if aPL persistent positivity in the HD population is an additive risk factor over uremia for endothelial dysfunction.

## 2. Materials and Methods

Study design and population: We performed a cross-sectional observational study. Institutional Review Board authorization was obtained from our local ethics committee (Ethics Committee of Brugmann University Hospital—reference number CE2023/149) on 17 October 2023, on accordance with the Declaration of Helsinki. Informed consent was obtained from all participants in the study.

We reviewed the medical records of the 178 prevalent HD patients of our dialysis department from 1 June 2024 to 31 December 2024, and we identified the patients with aPL persistent positivity according to the laboratory domain of the 2023 ACR/EULAR classification criteria [9]; who met the inclusion criteria for the study. To reduce the influence of variables known to influence the FMD, and therefore minimize confusion bias, we matched each aPL-positive patient to an aPL negative control, according to age, gender, diabetes mellitus, cigarette smoking, and markers of dialysis adequacy (Kt/V).

Inclusion criteria were as follows: prevalent HD patients with thrice weekly HD (4 h) schedule, age > 18 years, HD vintage of at least 3 months.

The exclusion criteria were as follows: The absence of available aPL assay or uninterpretable assays (concomitant anticoagulant therapy, inflammatory state, or acute thrombosis and patients experiencing aPL negativization over time), the presence of an acquired or innate thrombophilia other than aPL, acute inflammation, neoplasia, hepatitis virus infection and any other severe cardiovascular complication (e.g., myocardial infarction, stroke, heart failure), infection, or surgery in the in the past 3 months, patients taking medications that could interfere with study results (e.g., anticoagulants, phosphodiesterase-5 inhibitors, β1-selective β-blockers, nitrites, α-1 adrenergic blockers, erythromycin, and protease inhibitors). Also, patients with AVF in both arms were excluded, whether it was functional or not.

Study groups: We classified patients according to the 2023 ACR/EULAR classification criteria for APS (Table 1) [9].

Patients were evaluated for the 6 clinical domains (macrovascular venous, macrovascular arterial thrombosis, microvascular involvement, obstetric complications, cardiac valve and hematological involvement) and the laboratory domain. A clinical and laboratory score was made according to the 2023 AC/EULAR classification. Patients were divided into the following groups:

- aPL+: gathering, APS patients (with at least 3 points in both clinical and laboratory criteria, and “aPL asymptomatic carriers” (with at least 3 points in laboratory criteria but less than 3 points in clinical criteria). APS and aPL asymptomatic carriers have been studied separately in a subgroup analysis.
- aPL−: patients with negative aPL assay, or patients with an initial positive assay but showing a negative assay at 12-week follow-up.

aPL assays: Only aPL assays performed in the past 3 years before the study were considered as suggested by the ACR/EULAR classification criteria [9]. The persistent positivity of aPL was defined by the positivity of one or multiple aPL on two or more occasions at least 12 weeks apart.

aPL assays are routinely performed before the first HD session of the week (i.e., 68 h gap between two hemodialysis sessions), on a yearly basis. Lupus anticoagulant (LA) was assessed by using a three-step diagnostic procedure: screening, mix and confirmation procedures using diluted-Russell-viper venom (dRVVT-Siemens^®^, Muenchen, German) and Silica Clotting time (SCT-Werfen^®^ Barcelona, Spain). LA was confirmed if one of the two functional coagulation assays (dRVVT or SCT) was positive in terms of Screening to confirmation ratio, using a citrated plasma sample (3.2%). The determination of anti-cardiolipin antibodies (aCL) and anti-β2 Glycoprotein I (aβ2-GPI) was performed by a chemiluminescence immunoassay (HemosIL Acustar aCL IgM/IgG Kit and aβ2-GPI IgM/IgG kit-Werfen^®^). Results were considered positive if IgG or IgM titers are >20 U/mL (corresponding to the 99th percentile).

Data collection: Patients’ demographic data, data on ESKD, as well as comorbidity conditions and treatments were obtained from patients’ medical records.

FMD measurement: FMD tests were performed using a standardized protocol by one experienced nephrologist (MT) blinded to the patient’s aPL status in order to limit the measurement bias. Each patient underwent a measurement of FMD on brachial artery (5 cm above the antecubital fossa) of the arm opposite the AVF, by high resolution ultrasound imaging (Marque, 5–10 MHz linear probe). Each test was performed after 15 min of resting in supine position, in a temperature-controlled room. All vasoactive medications were withheld 12 h before the procedure, and examination was performed after overnight fasting, before the first HD session of the week (68 h gap between two hemodialysis sessions). Also, patients were asked not to smoke or consume caffeine at least 12 h before FMD test.

**Figure 1:**
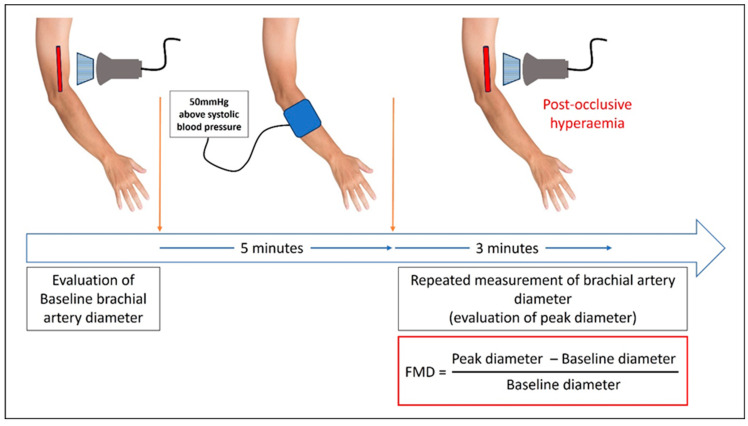
Procedure used for FMD of the brachial artery measurement. FMD: flow-mediated dilation.

Longitudinal images of brachial artery were taken, and baseline diameter of the brachial artery was obtained as the mean of three consecutive measurements. Then a sphygmomanometer was inflated for 5 min, 50 mmHg above the systolic pressure (no more than 250 mm Hg). Peak diameter was obtained by repeated measurements every 30 s for 3 min after the cuff was released. Results were expressed as the percentage change from baseline diameter of brachial artery (Figure 1).

Dialysis prescription: All patients were dialyzed with bicarbonate-based dialysate, single-use synthetic dialyzers (polysulfone) and low molecular weight heparin as standard anticoagulant. All patients were on a thrice weekly HD (4 h) schedule. Dialysis prescription was guided by a goal of achieving a value of Kt/V ≥ 1.4.

Statistical analysis: The statistical analysis plan was established before the collection of data. Data are presented as mean ± SD for normally distributed variables or median (25th–75th percentile) for non-normally distributed variables, or absolute or relative frequencies for nominal variables. We compared the group of patients with positive aPL with the group with negative aPL (controls). The statistical comparison was made using t test for variables with normal distribution or the Mann–Whitney rank sum test for the others. Paired *t*-test was used to evaluate quantitative predictive and outcome variables and X^2^ test for qualitative ones. *p* ≤ 0.05 was considered statistically significant. Statistical analysis was performed using SPSS software, version 31.

## 3. Results

**Figure 2:**
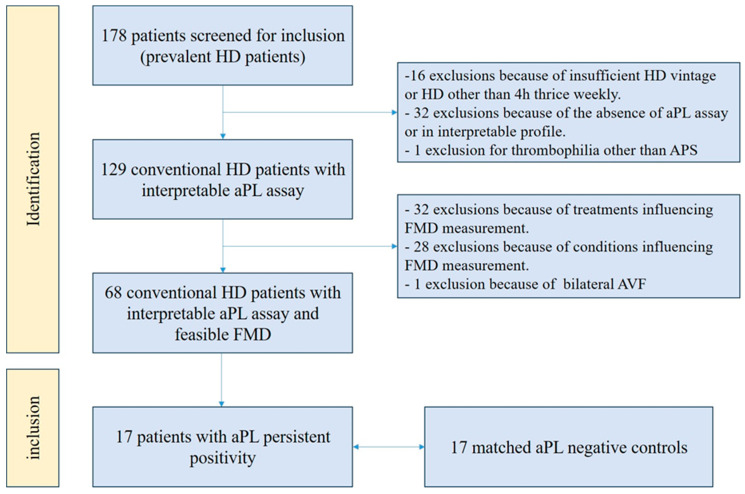
Study flow chart. aPL: antiphospholipid antibody; APS: antiphospholipid syndrome; AVF: arteriovenous fistula; FMD: flow-mediated dilation; HD: hemodialysis.

Out of 178 prevalent HD patients in our dialysis department, 17 patients with aPL persistent positivity met the inclusion criteria and were included (study flow chart is presented in Figure 2). Seven patients were classified as APS and 10 patients as aPL carriers without APS. With respect to aPL profiles, 16 patients had single LA persistent positivity, whereas 1 APS patient had double LA and IgM aCL positivity. No patient had concomitant autoimmune disease.

Baseline characteristics and FMD values according to aPL status are presented in Table 2.

Data are presented as arithmetic mean and standard deviation (SD) in normally distributed variables and in median and interquartile range (IQR) in asymmetric variables.

Definitions are as follows: ACEi, angiotensin-converting enzyme inhibitors; ARB, angiotensin II receptor blockers; BMI, body mass index; HD, hemodialysis; HDF, hemodiafiltration; TIA, transient ischemic attack.

The two groups were equally distributed in terms of demographic parameters, and medical history and had similar cardiovascular burden. Also, there was no difference in terms of treatment between groups.

**Figure 3:**
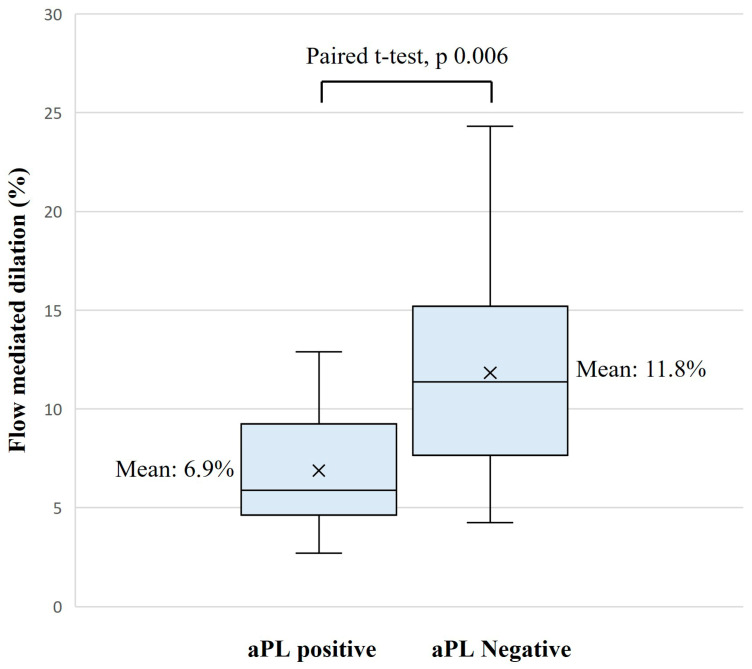
Box plot comparison of FMD between aPL-positive patients (n = 17) and controls (n = 17). The line inside the box plot represents the median. The cross within the box plot represents the mean. aPL: antiphospholipid antibody.

Results of FMD tests were significantly lower in the aPL-positive group, with a mean (SD) of 6.9% (2.8) vs. 11.8% (5.5), mean difference (IC 95%): −4.9 (−8.3; −1.6), *p* = 0.006 (Table 2, Figure 3).

**Figure 4:**
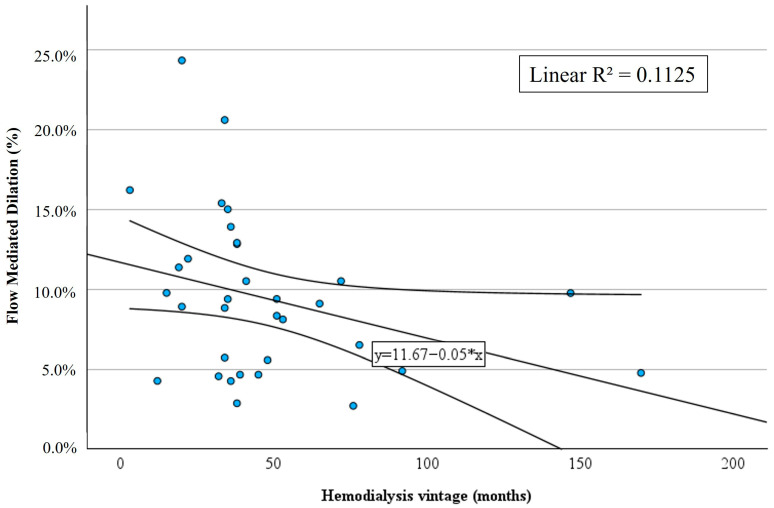
Flow-mediated dilation of the brachial artery according to patient’s hemodialysis vintage.

In relation to HD parameters, patients in the aPL-positive group had a longer HD vintage with a median of 51 months in the aPL-positive group vs. 35 months in the control group, mean difference (IC 95%): −27.1 (50.2; −3.9), *p* = 0.023 (Table 2). We observed a trend to a lower FMD for patients with longer HD vintage with a linear R^2^ of 0.1125 (Figure 4). HD adequacy was similar between groups in terms of Kt/V and pre-HD urea, and modality of dialysis was similar between groups with 41% of patients being on HDF vs. 59% on HD in both groups (Table 2).

With respect to laboratory findings, patients in the aPL-positive group had a slightly higher c-reactive protein level, with a mean (standard deviation) of 5.2 mg/dL (6.3) and 14.2 mg/dL (14.2), mean difference (IC 95%): −9.0 (−17.2; −0.8), *p* = 0.032.

In multivariable analysis adjusting for CRP, and HD vintage, aPL positivity remained associated with a meaningful but statistically borderline reduction in FMD (6.514 percentage point reduction in FMD, *p* = 0.077), possibly due to limited sample size (Table 3).

## 4. Discussion

Endothelial dysfunction has been well described in HD patients, and uremia contributes to the latter [21,22]. This cross-sectional study aimed to evaluate the possible additional role of aPL positivity over uremia in the pathophysiology of endothelial dysfunction in HD patients. To our knowledge, we report for the first time a more severe endothelial dysfunction with a lower FMD in aPL persistently positive HD patients. Endothelial dysfunction is a key element in the pathophysiology of APS. Indeed, aPL are directed against phospholipid and phospholipid binding protein leading to immune complexes that can trigger endothelial cells. APS patients exhibit lower nitrites and nitrates level, which are the stable metabolites of NO breakdown and lower FMD is found in APS patients and eNOS inhibition-mediated endothelial dysfunction have been suggested as a first hit in the pathogenesis of APS, ultimately leading to clinical manifestations [6,10]. eNOS) inhibition has been shown to be dependent upon protein phosphatase 2A (PP2A), β2-GPI, and apolipoprotein E receptor 2 [6], however, other mechanisms have been described such as Asymmetric Dimethylarginine-mediated eNOS inhibition or impaired paraoxonase activity leading to oxidative stress and endothelial dysfunction [12,13,14]. However, these pathophysiological mechanisms have not been studied in HD population.

Endothelial dysfunction is considered as a marker of vascular complications and cardiovascular burden, especially in ESKD patients [21,23]. It has been associated with increased oxidative stress and inflammation factors recognized to be involved in the pathogenesis and the progression of atherosclerosis in the early stages [24,25]. There was no difference between groups in terms of hypertension, stroke or TIA, ischemic heart disease. In our study, each aPL-positive patient was matched with a control for factors known to influence FMD (i.e., age, gender, diabetes mellitus, cigarette smoking and marker of dialysis adequacy (Kt/V)). We found a trend toward higher prevalence of peripheral artery disease in the control group. Therefore, the difference in terms of endothelial dysfunction between groups is not related to cardiovascular burden in this study.

Some data suggest that ESKD and cardiovascular disease are independently associated with endothelial dysfunction [3]. Actually, ex vivo study on cultured endothelial cells exposed to sera of HD patients showed that inflammasome proteins such as TLR4 and NALP3 contribute to the development and perpetuation of endothelial dysfunction in response to the uremic toxicity [22]. Also, there are possible implications of dialysis modality and HD membranes on endothelial dysfunction, as they influence the efficiency of inflammatory cytokines and large, middle-sized uremic toxins removal [26]. However, there was no difference in terms of dialysis membrane, nor in terms of uremic parameters between groups.

Thrombosis has also been linked to endothelial dysfunction in APS patients [15]. We did not find an association between FMD and venous or arterial thrombotic manifestations.

Interestingly, CRP level was found to be significantly, however slightly higher in the aPL-positive group, which was not explained by difference in dialysis adequacy and uremic parameters. This association has been reported by others, and APS, like other autoimmune diseases can be considered as an inflammatory disease [13,27]. CRP has been associated with decreased eNOS function and reduced angiogenesis in this population [28]. Interestingly, β2-2GPI/anti-β2-2GPI antibodies complexes have been shown to activate endothelial cells through the TLR4/MyD88 pathway, and ultimately NF-kB resulting in the expression of inflammatory genes in endothelial cells [6,10].

In addition, we found that aPL-positive patients had a longer HD vintage, which has been reported by others [29], hypothesizing that chronic exposure to HD membranes, to endotoxins, to oxidative stress, triggers the onset of aPL [16]. Interestingly, in our cohort, we observed a trend toward lower FMD in patients with longer HD vintage, which has not been described in the literature. Whether chronic exposure to HD leads to the stimulation of aPL formation and to more severe endothelial dysfunction has to be determined in further study.

In multivariable analysis adjusting for CRP, and HD vintage, aPL positivity remained associated with a meaningful but statistically borderline reduction in FMD (6.514 percentage point reduction in FMD, *p* = 0.077), possibly due to limited sample size. Despite the slight difference between groups in terms of CRP (10 mg/dL vs. 3 mg/dL; both within normal laboratory values), CRP seems to be a confounding factor. This should be confirmed in further studies. Whether higher CRP is related to the aPL status or in associated with another condition needs to be further evaluated in other studies.

In our study, only aPL profiles with low risk were included as patients treated with anticoagulants were excluded. Indeed, anticoagulant therapy might influence endothelial function. Direct oral anticoagulants such as apixaban have been shown to increase vasodilation in patients treated for atrial fibrillation [30]. Moreover, in experimental in vitro models of endothelial dysfunction resulting from uremic toxins, apixaban has been shown to upregulate expression of eNOS [31]. To our knowledge, there is no specific data on endothelial dysfunction and warfarin. However, few studies have compared apixaban to warfarin and showed similar endothelium-dependent vasodilation by using FMD [32]. Therefore, we decided to exclude patients treated with anticoagulants, as the purpose of the study was to evaluate the potential role of aPL over uremia in terms of endothelial dysfunction in HD patients.

The present study has several limitations which limit the generation of hypothesis. Firstly, the sample size is small, which limits the statistical power (risk of type II error) and generalizability of the results to broader populations. Secondly, due to the cross-sectional design, only association can be described and no causal relationship between aPL positivity and endothelial dysfunction can be established in this population. Also, this design prevents evaluation of temporal relationships, such as whether aPL positivity precedes the development of endothelial dysfunction or vice versa. Despite the study design that aimed to strengthen internal validity and to minimize confounding factors, residual confounding from unmeasured variables may still be present such as lifestyle factors or genetic factors. Also, patients receiving anticoagulant therapy were excluded, which may limit the generalizability of our findings. Finally, FMD measurements were performed manually and were operator dependent.

Future studies with larger sample sizes, more comprehensive data collection, and longitudinal or interventional designs will be needed to address these limitations and clarify causal pathways. We are currently conducting an observational cohort study designed to assess the association between aPL positivity and arteriovenous fistula complications, including maturation failure. A minimum of 100 patients are expected to be enrolled. All participants will undergo aPL assays and FMD measurements at the same timepoint (CKD stage G5, prior to initiation of HD and before AVF creation), which will provide valuable data for evaluation in terms of HD vintage, also, it will provide valuable longitudinal data. The full study protocol has been previously published [33].

## 5. Conclusions

We describe an association between aPL persistent positivity (mostly LA) and endothelial dysfunction with lower FMD, independent of classical known risk factors. In our HD population, aPL positivity was associated with higher c-reactive protein and HD vintage. These findings must be confirmed in a larger cohort, considering the HD vintage as a potential confounding factor.

## Figures and Tables

**Table 1:** 2023 ACR/EULAR classification criteria for antiphospholipid syndrome.

	2023 ACR/EULAR
Classification	3 points from clinical domains AND at least 3 points from laboratory domains LA IgG, IgM aCL IgG, IgM aβ2GPI Entry criteria and scoring
Clinical criteria	6 clinical domains 1. Macrovascular-Venous Thromboembolism 2. Macrovascular-Arterial Thrombosis 3. Microvascular 4. Obstetric 5. Cardiac Valve 6. Hematology
Laboratory criteria	
Persistent positivity (12-week confirmation assay)	Yes
Timeline of aPL positivity and clinical criteria	Within 3 years of clinical criterion
Antibodies for laboratory criteria	Isolated IgM aCL and/or IgM aβ2GPI are not sufficient (weight only 1 point)

aCL: anticardiolipin antibody; aβ2GPI: anti-β2 Glycoprotein I; LA: lupus anticoagulant.

**Table 2:** Patients’ baseline characteristics and FMD values according to aPL status.

Characteristics	aPL-Positive (*n* = 17)	Controls (*n* = 17)	*p* Value
Demographics			
Age, years, mean (SD)	58.5 (16.3)	60.8 (14.9)	0.671
Gender, male, n (%)	10 (59)	10 (59)	-
BMI, kg/m^2^, mean (SD)	27.7 (7.0)	27.1 (5.6)	0.814
Smoker, n (%)	2 (12)	2 (12)	-
Medical history (n (%))			
Diabetes mellitus	10 (59)	10 (59)	-
Hypertension	17 (100)	17 (100)	-
Stroke	3 (18)	3 (18)	-
TIA	1 (6)	1 (6)	-
Ischemic heart disease	6 (35)	5 (29)	0.724
Peripheral vascular disease	4 (24)	7 (41)	0.234
Deep vein thrombosis	2 (12)	2 (12)	-
Dialysis modality			
HDF/HD, n (%)	7 (41)/10 (59)	7 (41)/10 (59)	-
HD vintage, months, median (IQR)	51 (41)	35 (17)	0.023
Urea Kt/V	1.6 (0.4)	1.5 (0.3)	0.358
Pre-dialysis urea, mg/dL, mean (SD)	124 (28)	120. (30)	0.747
Urinary output (>500 mL), n (%)	5 (29)	5 (29)	-
Laboratory			
Hemoglobin, g/L, mean (SD)	11.5 (1.5)	11.5 (1.3)	0.981
Platelet, ×10^3^/µL, mean (SD)	233 (74)	226 (54)	0.770
Mean platelet volume, fl, mean (SD)	10.1 (0.9)	10.4 (0.9)	0.380
APTT, seconds, mean (SD)	27 (5.8)	26.(4.3)	0.806
c-reactive protein, mg/L, median (IQR)	10 (14)	3 (5)	0.032
Treatments			
Low-Dose Apirin, n (%)	9 (53)	13 (77)	0.161
Statins, n (%)	7 (41)	12 (71)	0.089
Erythropoietin, n (%)	14 (82)	14 (82)	-
Erythropoietin, UI/Kg (SD)	48 (33)	55 (50)	0.601
ACEi/ARBs	10 (59)	7 (41)	0.159
β-blockers	13 (77)	10 (59)	0.143
Flow-mediated dilation			
Brachial artery diameter, mm, mean (SD)	0.4 (0.05)	0.4 (0.05)	0.266
Flow-mediated dilation, mm, mean (SD)	6.9 (2.8)	11.8 (5.5)	0.006

**Table 3:** Factors associated with FMD (dependent variable), in multivariable linear regression.

	Multivariate Linear Regression Model			
Independent Variables	B	OR	95% CI	*p* Value
C-reactive protein	0.364	0.032	−0.04–0.767	0.055
Hemodialysis vintage	0.039	0.008	−0.067–0.145	0.135
aPL positivity	−6.514	0.787	−16.517–3.489	0.077

## Data Availability

The datasets used and/or analyzed during the current study are not open access.

## Abbreviations

The following abbreviations are used in this manuscript:

aPL	Antiphospholipid antibody
aCL	Anti-cardiolipin antibody
APS	Antiphospholipid syndrome
aβ2-GPI	Anti-β2 Glycoprotein I antibody
CRP	c-reactive protein
eNOS	Endothelial Nitric Oxide synthase
ESKD	End-stage kidney disease
FMD	Flow-mediated dilation
HD	Hemodialysis
LA	Lupus anticoagulant
NO	Nitric oxide
